# Parents’ and Children’s Categorization of Candy are Similar: A Card Sort Protocol

**DOI:** 10.3390/nu11102472

**Published:** 2019-10-15

**Authors:** Jennifer S. Savage, Holly A. Harris, Julia A. Bleser, Brandi Y. Rollins

**Affiliations:** 1 Center for Childhood Obesity Research, The Pennsylvania State University, 129 Noll Laboratory, University Park, PA 16802, USA; hah5311@psu.edu (H.A.H.); jat382@psu.edu (J.A.B.); byr104@psu.edu (B.Y.R.); 2 Department of Nutritional Sciences, The Pennsylvania State University, 110 Chandlee Laboratory, University Park, PA 16802, USA

**Keywords:** card sort, candy intake, children, parents, adults, sweet snacks, obesity prevention

## Abstract

American children frequently consume candy and, in excess, this may contribute to poor diets with attendant effects on obesity risk. Despite the ubiquity of candy in children’s diets, parental concern about children’s candy intake, and the diversity of confectionery products available, very little is known about how children and their parents conceptualize candy. Card sorting tasks offer a novel and visual technique to explore and compare an individuals’ perceptions of foods and are useful where literacy is limited (e.g. young children). This study aimed to understand and compare how young school-aged children and parents categorize various candy products using a photo card sorting task. In individual laboratory sessions, children (*n* = 42, 5 to 8 years old) and parents (*n* = 35) categorized 51 types of candy based on their similarity. A cluster analysis showed that parents created more categories of candies than children (11 versus 8). For example, parents distinguished between candied fruit and candied nuts, whereas children tended to collapse these categories. However, 7 clusters were virtually identical between parents and children (93% similarity). The findings from this study can inform the measurement of candy intake and the development of education materials targeted towards parent feeding around candy.

## 1. Introduction

Candy is the fifth largest contributor to children’s energy intake from added sugar [1,2], with almost one third (31%) of American children consuming candy daily [3]. The largest increases in snacking events over the past three decades have been in candy and salty snack food consumption [4]. The terms used to describe candy, sweet confections and chocolate are ambiguous and vary widely across the world. In the United States (USA) and Canada, ‘candy’ typically describes sweet confectionary food with sugar or sugar substitutes as its principal ingredient. However, sweet confectionary food is termed ‘sweets’ in the United Kingdom (UK) and ‘lollies’ in Australia and New Zealand [5]. The current paper refers to candy to describe sugar confectionary (including hard candy, sugar candy and lollies) and chocolate confectionary, as characterized by Adams and Savage [6]. A defining characteristic of candy is its sweet taste which appeals to children’s innate preference for sweet-tasting foods [7]. This raises concern for parents, who may struggle in regulating their children’s candy intake, despite attempts to restrict or limit access [8]. 

Despite the ubiquity of candy in children’s diets, parental concern about children’s candy intake, and the diversity of chocolate and non-chocolate candy available in the market, the current measures of candy intake are very limited. For example, food frequency intake measures used in epidemiological research often rely on 1–2 questions to assess chocolate and non-chocolate intake [9]. However, these questions may fail to prompt participants to recall the different types chocolate and non-chocolate candies they may have consumed. Without an understanding of how people identify different types of candy, it is unclear whether candy intake may be under-reported or overreported in response to food frequency questionnaires. As such, trends in candy consumption may be inaccurate [1,2,3,4], thus affecting the ongoing monitoring and evaluation of national dietary patterns. Food intake measures can be informed by understanding how school-aged children (i.e., children attending elementary or primary between the ages of 5 and 11 years old) and their parents conceptualize and categorize chocolate and non-chocolate candy. Additionally, researchers aiming to develop materials that target children’s intake of candy would benefit from understanding how parents and children categorize the diverse selection of candy available on the market. This is the aim of the current study. 

The association between candy consumption and child adiposity parameters is mixed. Children’s consumption of sweets, including candy, have been positively associated with adiposity [2], while other studies report negative [10,11,12] or null [13] relations. This inconsistency may be due to the lack of an operational definition of candy and varied approaches for measuring the composition and frequency of eating occasions [14,15]. This is demonstrated in National Health and Nutrition Examination Survey (NHANES) data listing candy in 6 different MyPyramid groups [16]. A better understanding of how school-aged children and their parents describe and categorize candy may help improve intake measures in epidemiological work. This understanding may also inform the development of screening tools and educational resources which use consistent terminology to describe candy. 

The aim of the current study was to use a card sorting technique to understand how school-aged children and their parents conceptualize and categorize a range of sweet-tasting candy products. A secondary aim was to qualitatively describe the similarities and differences between children and adults’ categorization of candy. Card sorting methods allows researchers to study how people organize and categorize their knowledge [17]. Card sort techniques have been successfully used with children and adults to describe their categorizations of other food groups including mixed foods, fruits and vegetables, and grain products [18,19,20,21,22]. Card sorts are appropriate for young children because photographs can be used and literacy is not required. Children aged 5 to 8 years old have demonstrated dexterity in card sort tasks to distinguish between snack foods and candy [6]. 

## 2. Materials and Methods 

### 2.1. Subjects

All parents and children were recruited through flyers posted at local schools and preschools, and via website advertisements. The inclusion criteria required that parents have a child between 5 and 8 years old who consumed candy at least once a month. Child participants were excluded if they had a health condition that could affect food intake or had known food allergies. Thirty-five families were initially recruited to participate in a parent focus group on a university campus to discuss parents’ perceptions and management of candy at home. Three months after completing the focus groups, the parents were invited to have their 5 to 8 years old child participate in a one-off laboratory session, in which children’s perceptions and intake of candy were measured. An additional 23 parent-child dyads were recruited to participate in this “child session”. Among the 58 parent-child dyads recruited, 35 parents (34 mothers, 1 father) completed the “parent session” and 42 children (20 boys, 22 girls; mean ± standard deviation, 7.1 ± 1.0 years old) completed the child session. The sample sizes for each group met the minimum recommended number of 10-15 participants to approximate similarity matrices [23]. The families were compensated for their participation ($25 for the parent session, $50 for the child session; families could receive up to $75 in compensation). The procedures were approved by and conducted in accordance with standards of the Pennsylvania State University Institutional Review Board. The parents provided informed consent and children’s verbal assent was obtained prior to the start of the testing session.

### 2.2. Card Sort Task

**Figure 1:**
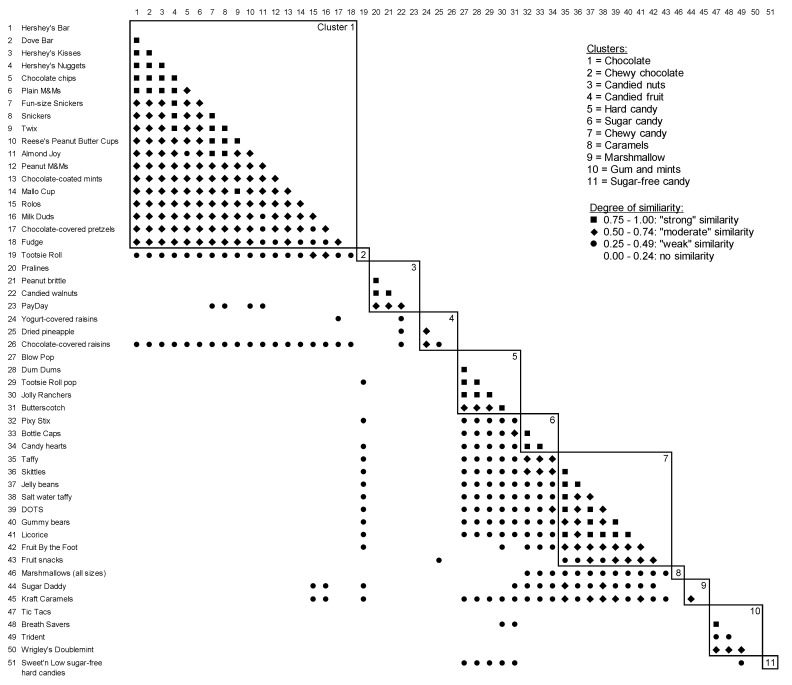
Similarity matrix of parent’s card sort of 51 candy items into piles (*n*= 35).

A card sort task similar to that previously used to measure 8 to 13 years old children’s categorizations of grains [19], and fruits and vegetables [21] was used in the current study to assess parents’ and 5 to 8 years old children’s categorizations of candy. Ultimately, this informed the development of measurement around candy intake and feeding practices, and intervention protocols for a larger study. The card sort instructions were simplified to make them developmentally appropriate for children as young as 5 years-old. National consumer purchasing data obtained from the National Confectioners’ Association (NCA) and candy food codes used in NHANES 2009-2010 [24] were reviewed and used to compile a list of common types of candy (e.g. full-size chocolate bars, fruit-flavored chewy candies, lollipops, chewing gum). Senior researchers on the study team and industry experts reviewed these data and codes, and a selection of 51 types of candies (shown in Figure 1) were chosen to represent a variety of common candy types.

In the parent session, parents were given a stack of 51 cards (4.5 × 5.5 inches), each displaying the name and colored photo of a specific type of candy (e.g. lollipop, chocolate bar). The parents were asked to sort the cards into piles based on similarities among the candies. The parents were assured there were no right or wrong answers. The parents began by comparing the first and second card in the deck, and asked to place these cards together if they felt they were similar or to start a new pile if they felt it was different. The parents were asked to create at least 4 piles and piles containing one card were allowed. They were told to not sort based on liking or how frequently the candies were consumed. The parents were instructed to put any pictures of foods they did not recognize in a “Don’t know” pile and that if they were not sure where to place a food, to create a “Not sure” pile. When the parents were finished sorting all of their cards, they were asked to look through the Don’t know and Not sure piles to determine if the cards could be re-sorted into the other piles. If not, the cards were kept in the Don’t know and Not sure piles. Once all of the cards were sorted, each parent was asked to write down a label and description for each pile on index cards. 

The children completed the card sort task in a one-on-one child session with a trained interviewer. Prior to the card sort task, each child spent time with the research staff during a meal, playing board games and completing craft activities. Each child was told “Here’s a stack of cards. Can you create piles of the foods that you feel go together? You can make as many piles or as few piles as you want.” There was no minimum requirement of piles for children to make. The children were reassured that there were no right or wrong answers. The children were instructed to give the interviewer any cards with candies they were unfamiliar with or did not know where to put them. The interviewer placed these cards in the Don’t know or Not sure piles, respectively. They were also told that they could change their mind and move the cards around as much as he or she liked. Once the child completed the card sort, the interviewer asked the child if he or she could re-sort any of the cards in the Don’t know or Not sure piles into the other piles. Next, the interviewer selected the largest pile, spread out the cards so that the child could see each card, and asked the child “What would you name this pile of cards? If you could give the pile one name, what would it be?” Once the child named the pile, they were asked “Why did you give the pile that name? What makes the cards in this pile alike?” The interviewer recorded the child’s responses. 

### 2.3. Study Demographics

The parents were asked to complete a brief demographic survey at the start of the research sessions asking about parents’ educational attainment, marital status and household income. Parent body mass index (BMI, weight (kg)/height (m)^2^) scores were generated based on parents’ self-reported height and weight. The parents were classified as underweight (BMI < 18.5 kg/m^2^), normal weight (BMI ≤ 24.9), overweight (BMI ≥ 25), or obese (BMI ≥30) [25]. The children’s height and weight were measured in triplicate by research staff in the laboratory during their child visit. The children’s BMI percentiles were computed using the 2000 CDC growth charts [26], and children were classified as overweight (BMI percentile > 85th and < 95th) or obese (BMI percentile ≥ 95th).

### 2.4. Data Analysis

Analysis of variance (i.e., ANOVA) was conducted in the Statistical Package for the Social Sciences (SPSS) software version 21 (IBM Corporation, Armonk, NY, USA) to determine if the quantity of candy piles created by the participants differed by individual parent and child characteristics. The participant’s card sort data were analyzed separately for parents and children using SynCaps software version 3 (Syntagram, Oxfordshire, UK). Two similarity matrices were exported from Syncaps, one for the parent card sort and another for the child card sort. A similarity matrix displayed the percentage of times (between 0 and 1.00) each possible pair of items appeared together in the same pile across the sample of participants. For example, a similar score of 0.85 would indicate that 85% of the time, two specific candies (e.g. chocolate chips and chocolate bars) were placed in the same pile. The similarity scores between 0.75–1.00 indicate a “strong” similarity, scores between 0.50–0.74 indicate a “moderate” similarity, scores between 0.25–0.49 indicate a “weak” similarity, and scores less than 0.25 indicate no similarity [27]. An average maximum similarity score for the parent card sort data was computed by averaging the similarity scores across all the candy pairs. For example, a 1.00 average maximum similarity score would indicate that parents created exactly the same set of piles, whereas a score of 0 would indicate that parents created completely different piles. Using the same approach, an average maximum similarity score was computed for the child card sort data. The outliers were also evaluated within SynCaps to determine if one or more participants created piles that were substantially different from other participants. However, no outliers were identified in the parent or child card sort data. 

To determine the number of candy clusters or categorizations for each card sort, the similarity matrices were imported into the SPSS Statistics software version 21 (IBM Corporation, Armonk, NY, USA) and a two-step hierarchical cluster analysis using Ward’s method was performed separately for the parent and child data [28]. In this clustering method, each candy type was paired with the next most-similar candy until the clusters formed with equal dissimilarity between the newly formed clusters. The ideal number of groups was quantified using the change in eigenvalues [29]. Next, *k*-means clustering was used to re-sort the items into the appropriate number of clusters. To provide potential names for the clusters, the parents’ pile names were compiled and standardized. For example, if one participant labeled a pile “sugar candy” and another participant labelled a pile “sugary,” these piles were re-labeled as “sugar candy.” A research assistant first compiled the pile names and two senior members of the research team reviewed the new labels for accuracy and resolved any discrepancies. The labels were then used to name the identified clusters.

## 3. Results

### 3.1. Preliminary Results

The parents (*n* = 35) were predominately white (94%) and non-Hispanic (94%), with a self-reported BMI of 24.9 ± 5.3 kg/m^2^ and 43% were identified with overweight or obesity. The majority of the parents were married (86%), middle- to upper-income (median: $60,000–$80,000), and well-educated (89% had at least a college degree). The children who participated in the child sessions (*n* = 42) had a similar socio-demographic profile and BMI percentile of 44.0 ± 29.1 and 12% of children were identified with overweight or obesity.

### 3.2. Parents’ Card Sort

The parents formed 7.9 ± 1.8 piles, with each pile containing 6.9 ± 1.7 cards. The number of piles created was not significantly associated with any sociodemographic characteristics (*p*’s > 0.05; data not shown). Based on the overall similarity matrix, the parent card sort data had an average maximum similarity of 0.75 ± 0.11 (possible range: 0 to 1.00). A cluster analysis yielded 11 clusters of candy categorizations (Figure 1) with eigenvalues exceeding 1.0: chocolate, chewy chocolate, candied nuts, dried fruit, hard candy, sugar candy, chewy candy, caramels, marshmallows, gum and mints and sugar-free candy. Figure 1 displays the similarity matrix from the parent card sort data and indicates the degree of similarity (0 to 1.00, expressed as percentages) between each pair of the 51 candy types, organized by cluster. For example, 86% of parents agreed that Hershey’s chocolate bars and chocolate chips should be categorized together (strong similarity), 74% agreed that Hershey’s chocolate bars and Peanut M&Ms should be categorized together (moderate similarity), 25% agreed that Hershey’s chocolate bars and chocolate covered raisins should be categorized together (weak similarity) and 0% agreed that Hershey chocolate bars and candied walnuts should be categorized together (no similarity).

### 3.3. Children’s Card Sort

**Figure 2:**
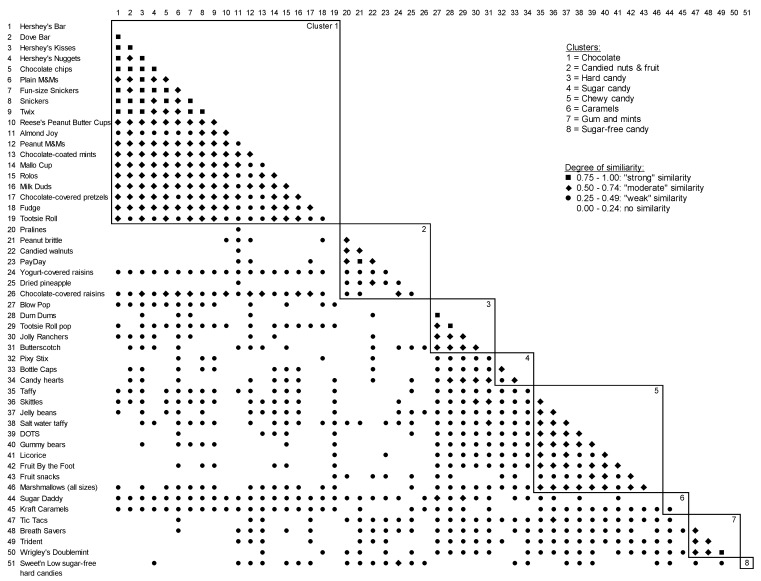
Similarity matrix of children’s card sort of 51 candy items into piles (*n* = 42).

On average, the children formed 5.9 ± 4.5 piles, with each pile containing 13.0 ± 7.9 cards. The number of piles created was not significantly associated with any sociodemographic characteristics (*p*’s > 0.05; data not shown). Based on the similarity matrix, the child card sort data had an average maximum similarity of 0.66 ± 0.11 (possible range: 0 to 1.00). A cluster analysis yielded eight clusters of candy categorizations (Figure 2) with eigenvalues exceeding 1.0: chocolate, candied nuts and dried fruit, hard candy, sugar candy, chewy candy, caramels, gum and mints and sugar-free candy. Figure 2 displays the similarity matrix indicating the degree of similarity (0 to 1.00, expressed as percentages) between each pair of the 51 candy types, organized by cluster. For example, 90% of children agreed that Hershey’s chocolate bars and chocolate chips should be categorized together (strong similarity), 57% agreed that Hershey’s chocolate bars and fudge should be categorized together (moderate similarity), 47% agreed that Hershey’s chocolate bars and chocolate covered raisins should be categorized together (weak similarity), and only 7% agreed that Hershey chocolate bars and candied walnuts should be categorized together (no similarity). 

### 3.4. Similarity and Dissimilarly Between the Parent and Child Card Sorts

There was a considerable overlap between the clusters identified in the parent and child card sorts (Figure 1 and Figure 2). Both the parents and child data generated 7 clusters that were nearly identical and were labelled similarly by parents and children, although they provided slightly different rationales for their labelling. To demonstrate, the following three examples of the labels and corresponding rationales are provided. The participants labelled the first category as chocolate; parents explained it had “candy that contains chocolate” while children explained “because everything has at least some chocolate”. The participants labelled another category as sugar candy. The parents explained that this category had “candy made of all sugar” while children explained that this candy has “lots of sugar in them”. Finally, the participants labelled another category as hard candy. The parents explained that “this candy is hard. You suck on it rather than chew it” while children explained “because they are all hard candies”. The remaining four similar clusters were chewy candy, caramels, gum and mints and sugar-free candy. 

Out of the 44 cards that comprised the 7 similar clusters, both the parents and children placed 41 of candy cards into identical clusters (93%). In other words, 41 cards or types of candy ended up in the same clusters based on both the parent and child card sort data. The parent and child data, however, showed small differences on three clusters. The parent data generated separate clusters for candied nuts and candied fruits, whereas the child data generated one combined cluster for candied nuts and fruit. However, when combined, they both consist of the same 7 cards (Figure 1 and Figure 2). In addition, the parent data generated a marshmallow cluster, containing one card for marshmallows, and a chewy chocolate cluster, containing one card for Tootsie Rolls. In contrast, children sorted these cards into other, less specific clusters (e.g. chewy candy or chocolate clusters). 

## 4. Discussion

The current study showed that school-aged children are able to categorize 51 types of candy in comprehensive ways which are similar to parents. While the majority of card sort categories were labelled and described similarly between parents and children, nuanced differences were evident. The parents tended to generate more categories of candy on average than children. This may indicate that parents have a more fine-grained quantification of candy based on observed characteristics or familiarity with the food. The parents also shared a stronger agreement within their categories of candy compared to children (0.75 versus 0.66 out of a possible 1.00), demonstrating slightly greater cohesion in perceptions of candy within the parents. This study provides initial evidence into how children and adults organize their thoughts as it relates to sweet confectionary foods which can better inform future nutrition interventions and measurement of candy intake. 

The parents and children created 7 similar categories of candy that closely mapped onto each other in terms of candy products selected (93% similarity), the labels provided and the justification of labels. The parents and children described these categories relating to salient characteristics (i.e., chocolate or gum and mints), and the taste and texture of the candy (i.e., hard versus chewy). The children rarely used the Don’t know or Not Sure pile when completing the card sorting task suggesting familiarity with these foods. Familiarity may be explained by children’s transition to school, as they are exposed to a wider array of foods outside the home environment, through peers, social occasions and marketing [30]. With greater exposure and familiarization to energy dense snack foods rich in solid fats and added sugars such as candy, parents may attempt to restrict children’s access and intake of those foods [31]. However, control-based feeding practices are not effective, and in fact have counter-intuitive effects on eating behavior [32]. Educating parents on alternatives to restriction could have important implications in reducing overeating and obesity risk [33]. The findings from the current study could be leveraged for such educational purposes. For example, the nuanced groups and corresponding labels of candies identified in this paper could inform tailored interventions designed to help children moderate their intake of candy. Researchers could develop a screening tool with the identified labels to assess the home availability and children’s intake of candy, and then optimize intervention messaging to focus on those types of candy. 

The categorization patterns revealed a greater degree of diversity of non-chocolate candies, with 31 of the 51 candy cards falling into non-chocolate categories such as hard candy, sugar candy, chewy candy, caramels and so forth. The children identified 7 non-chocolate categories and parents identified 9 non-chocolate categories, compared to 1 and 2 chocolate categories, respectively. The current food intake measures, such as the NHANES [34] or Harvard [9] food frequency questionnaires, fail to capture the diversity of non-chocolate candies. In another example, the Australian National Health Survey (NHS) [35] uses a 5-phase, 24-hour dietary recall, with one prompting phase asking participants to report on their intake of sweets: biscuits, lollies, ice cream, or other sweets. This protocol does not appear to prompt for the intake of specific categories of sweets/lollies, nor does it prompt for chocolate or chocolate-based confectionary. In addition, the NHS asks parents to report on behalf of or assist children in reporting their child’s intake between the ages of 6 to 11 years old [35]. Therefore, the current study findings add value in understanding how parent-proxy reports and self-reported child candy intake may vary. Without appropriate prompting, the participants may have difficulty in accurately recalling the many different types of chocolate and non-chocolate candies. This question merits further research into the potential of underreporting of candy. Further research could also examine the utility of providing additional candy categories on food frequency measures or for organizing candy categories in computerized dietary intake systems [17]. 

The parents created 3 clusters of candy that children did not identify as individual categories. Firstly, the parents distinguished between candied nuts and candied fruit while the children collapsed these categories. This may be in part explained by age- and developmental-related cognitive differences between adults and children. The parents also created two individual categories which consisted of individual cards in their own separate clusters: chewy chocolate (Tootsie Roll) and marshmallow (marshmallows all sizes). The adults may have distinguished marshmallows from other candy due to cooking characteristics associated with them (campfire s’mores or making them into Rice Krispies), whereas Tootsie Rolls may have a vintage or nostalgic connotation unrelated to other chocolate. Despite these differences, the results remain indicative that school-aged children are capable of understanding and classifying a large proportion of candies in a manner similar to adults. 

The study findings must be interpreted in light of its strengths and limitations. Detailed information was obtained about the parents’ and children’s categorization of candy, while other studies using card sort techniques tended to categorize across a range of food groups. The exposure to and intake of each type of 51 candies were not measured in the current study, and therefore assumptions cannot be made about the parent or child diet. The study was undertaken in a well-educated, mostly white, middle- to upper-income sample and the findings may not be generalizable to other sociodemographic groups. As family income and parent education may be associated with the number of categories children create to group foods [19], the current findings must be replicated in more diverse and larger samples that allow for sub-group analyses. 

In summary, the current study shows that young, school-aged children are capable of creating meaningful categories of candy. The children’s categories of candy broadly reflected those of the parents’, demonstrating similarity in cognitive structures underlying how candy is grouped together by parents and children. This has implications for both research and practice in terms of the measurement of dietary intake, and providing a comprehensive lexicon that researchers can use to develop intervention materials that target parenting around children’s candy intake.
